# MiR-371b-5p reduces osteosarcoma cell migration and proliferation to induce apoptosis by targeting FUT4

**DOI:** 10.7150/jca.103286

**Published:** 2025-04-13

**Authors:** Qiang Xue, Ruicong Ma, YueYuan Chen, Xiaodi Yan, Jiajia Liu, Jianhua Xue, Yang Yang, Xianchen Liu

**Affiliations:** 1 Department of Radiation Oncology, Affiliated Hospital of Nantong University, Nantong City, Jiangsu Province 226001, China.; 2 Department of Oncology, Second People's Hospital of Nantong & Affiliated Nantong Rehabilitation Hospital of Nantong University, Nantong City, Jiangsu Province 226001, China.; 3 Department of Trauma Center, Affiliated Hospital of Nantong University, Nantong City, Jiangsu Province 226001, China.; 4 Department of Chemistry, School of Science, China Pharmaceutical University, Nanjing City, Jiangsu Province 211198, China

**Keywords:** bone neoplasm, miRNA, oncoprotein

## Abstract

In our previous study (PMID: 34671604), we found that miR-317b-5b not only exerted anti-tumor effect, but also downregulated FUT4 expression in human myeloma cell line 143B. This study aims to investigate the biological function of miR-371b-5p in osteosarcoma progression and the role of FUT4 in this process. For *in vitro* investigations, the human osteosarcoma cell lines (SaOS2, 143B, KHOS and U2OS) as well as the human osteoblast cell line (hFOB1.19) were employed as models. The QRT-PCR assay was utilized to determine the relative amounts of miR-371b-5p and FUT4 expression in the cells. The functions and effects of miR-371b-5p on the abilities to proliferate, migrate, apoptosis and invade of KHOS and U2OS in osteosarcoma cells were investigated using assays including CCK-8, colony formation, EDU, wound-healing, Western blot, TUNEL and Transwell assay. The correlations between miR-371b-5p, its downstream gene FUT4 and its potential mechanisms in mediating osteosarcoma progression were explored with the assistance of dual-luciferase reporter analysis together with rescue experiments. MiR-371b-5p was less expressed in osteosarcoma cells compared with osteoblasts. Its overexpression significantly inhibited the abilities to proliferate, invade and migrate to promote apoptosis of osteosarcoma cells. The correlations between FUT4 and miR-371b-5p was established by the gene analysis of the dual-luciferase reporter analysis. FUT4 expression was dramatically decreased after the process of miR-371b-5p mimics being transfected into KHOS and U2OS cells. Additionally, overexpression of FUT4 induced osteosarcoma cell apoptosis and partially overcame miR-371b-5p's inhibitory effects on osteosarcoma cell's abilities to proliferate, invade and migrate. Osteosarcoma cells exhibit down-regulation of miR-371b-5p, that prevents osteosarcoma cells from proliferating, invading and migrating in order to promote osteosarcoma cell apoptosis through concentrating on the breakdown of FUT4.

## 1. Introduction

In children and teenagers, osteosarcoma, a kind of most prevalent primary malignant bone tumor, causes 8.9% of cancer deaths. It frequently affects the long-bone metaphysis, associated with the knee in more than 50% of cases 1. All osteosarcomas are extremely malignant, and 40% of clinical cases advance rapidly, showing recurrence and/or metastasis with less effective therapy 2. High-dose chemotherapy, surgery and neoadjuvant chemotherapy are currently the favored therapies for osteosarcoma. Despite improvements in diagnosis and threapy, the outlook for patients with osteosarcoma is remains grim, with a 5-year survival rate of little over 30% overall 3. Therefore, there is a critical need for novel molecular targets that will ultimately lead to efficient treatment approaches for osteosarcoma 4.

MiRNAs, as single-stranded non-coding RNAs, are between 18 and 25 nucleotides long 5, and it can impede mRNA translation or encourage mRNA degradation to control gene expression at the post-transcriptional stage. A few physiological processes and pathological outcomes, including cancer, are highly dependent on miRNAs 6. Studies have revealed that miRNAs have an appreciable effect on the development, differentiation, metastasis, apoptosis and angiogenesis of tumors 7, as a result of their ability to control whole signaling cascades rather than just specific proteins, miRNAs are intriguing therapeutic targets 8. In particular, miRNAs can be utilized as molecular therapies for cancer and other diseases 9. In our earlier research 10, it was discovered that synthetic exosomes of miR-371b-5p may reduce osteosarcoma cell 143B viability, proliferation, migration and invasion while promoting apoptosis. However, the expression pattern and regulatory mechanism of miR-371b-5p in osteosarcoma cells remain unclear. Interestingly, studies have found that miR-371b-5p is underexpressed in patients with adult T-cell lymphoblastic lymphoma 11 and triple-negative breast cancer 12, but overexpressed in NSCLC 13, playing a comparable role in driving the progression of tumors. Additionally, in 2015, S. Mutlu et al. extracted RNA from 18 decalcified surgical chondrosarcoma specimens for research and found no significant change in the expression of miR-371b-5p in chondrosarcoma specimens, although the results are questionable due to incomplete samples 14. There is still a significant gap in research regarding the role of miR-371b-5p in osteosarcoma.

Fucosyltransferase 4 (FUT4) is an oncoprotein 15 predominantly expressed in the bone marrow (https://www.proteinatlas.org/ENSG00000196371-FUT4/tissue). In our previous researches, when exosomes carrying miR-371b-5p were cocultured with human myeloma cell line 143B, the FUT4 levels decreased compared to the control group 10, compared to osteoblasts (hFOB1.19), osteosarcoma cells (KHOS and U2OS) expressed higher levels of FUT4, and inhibiting FUT4 expression reduced the proliferation, invasion, and migration abilities of osteosarcoma cells 15. Research by Cong Tian et al. found that osteosarcoma (OS) upregulates lncRNA MRUL, which promotes cell proliferation and metastasis by negatively regulating miR-125a-5p, and that lncRNA MRUL interacts with miR-125a-5p to inhibit FUT4 expression 16. Weijian Li et al. 's study on bladder cancer also shows that miR-371b-5p/FUT4 axis plays a role in tumor progression 17. These findings suggest that there may be an interaction between miR-371b-5p and FUT4 in osteosarcoma that contributes to tumor progression.

Current research has indicated that both miR-371b-5p and FUT4 play regulatory roles in osteosarcoma cells, and miR-371b-5p has also been found to affect the expression level of FUT4 in osteosarcoma cells. However, the mutual regulatory relationship and mechanism between miR-371b-5p and FUT4 in osteosarcoma still require further investigation. Therefore, this study intends to utilize human osteoblasts (hFOB1.19) as well as human osteosarcoma cell lines (SaOS2, 143B, KHOS, and U2OS) to explore the specific expression of miR-371b-5p in osteosarcoma cells. Through bioinformatics analysis and cellular experiments, we aim to study the interaction between miR-371b-5p and FUT4, and further investigate the regulatory role of miR-371b-5p/FUT4 in osteosarcoma cells. The goal is to initially validate the hypothesis that "miR-371b-5p regulates FUT4 to affect the progression of osteosarcoma".

## 2. Materials and methods

### 2.1 Cell culture

The American Type Culture Collection (ATCC) (Manassas, VA, USA) provided human osteoblasts (hFOB1.19, CRL-3602) as well as human osteosarcoma cell lines (SaOS2, HTB-85; 143B, CRL-8303; KHOS, CRL-1544; U2OS, HTB-96). A 5% CO_2_ incubator was used to incubate all of the cells at 37°C in Dulbecco's modified Eagle medium (DMEM) (11965092, Gibco, GrandIsland, NY, USA) with 10% fetal bovine serum (FBS) (A5670701, Gibco, GrandIsland, NY, USA), 1% streptomycin-penicillin (15140122, Gibco, GrandIsland, NY, USA).

### 2.2 Cell transfection

The day before transfection, cells were plated and inoculated. After cells had fused by 70-80%, they were transfected with reference to the guidances of Lipofectamine 2000 (11668500, Invitrogen, Carlsbad, CA, USA). After 4 h, the fresh medium was added. The mixture was then incubated for a further 24 h.

### 2.3 RNA extraction

In 1 mL of TRIzol (15596018CN, Invitrogen, Carlsbad, CA, USA), cells (5 × 10^6^ cells) were dissolved. 200 μL of trichloromethane was added following a five-minute incubation period at room temperature. It was then incubated at room temperature, being kept for 5 minutes. Then the supernatant was transferred to a fresh, ribonuclease-free centrifuge tube after 15 minutes of centrifugation at 4°C and 12,000 rpm. To collect the RNA precipitate by centrifugation, isopropanol was added along with the equal volume of supernatant. The isolated RNA was then dried by air and measured. Diethylpyrocarbonate (DEPC) (ST036, Beyotime, Shanghai, China), diluted in 10-20 μL, was used to dissolve the RNA samples.

### 2.4 QRT-PCR assay

Total RNA was extracted from cells and tissues by the TRIzol reagent. The quantity of RNA was determined using a UV spectrophotometer (Hitachi, Tokyo, Japan). The extracted RNA was then reverse-transcribed into complementary deoxyribonucleic acid (cDNA) by the PrimeScript^TM^ RT MasterMix kit (RR036A, Takara Bio Inc., Beijing, China). SYBR Green Master Mix (A46012, Applied Biosystems, Foster, CA, USA) was carried out in the quantitative real-time polymerase chain reaction (qRT-PCR) assay. The total volume of the qRT-PCR apparatus was 10 L. After 2 minutes of pre-denaturation at 95 °C, there were 40 periods of denaturation at 95 °C for 1 minute, 60 °C for 1 minute, and 72 °C for 1 minute. The relative expression levels of the target genes were determined using the method of 2^-ΔΔCt^. In Table 1, the primer sequences are displayed.

### 2.5 Dual-luciferase reporter analysis

To construct FUT4 WT and FUT4 MUT, respectively, miR-371b-5p combining site-containing wild-type (WT) as well as mutant (MUT) FUT4 sequences were introduced into the pGL3 dual-luciferase vector. Then miR-371b-5p mimic/negative control and FUT4 WT/FUT4 MUT were cotransfected into the cells. 24 h later, the transfected cells were lysed. For the measurement and determination of luciferase activity, a system of dual-luciferase reporter assay (E1960, Promega, Madison, Wisconsin, USA) was employed.

### 2.6 Western blot assay

Using a phenylmethylsulfonyl fluoride (PMSF) (ST505, Beyotime, Shanghai, China) and a radioimmunoprecipitation assay (RIPA) (P0013B, Beyotime, Shanghai, China), total proteins were recovered from cells. Before the protein samples were separated by 10% sodium dodecyl sulfate-polyacrylamide gel electrophoresis (SDS-PAGE) (P0012AC, Beyotime, Shanghai, China), the proteins were transferred to membranes (3010040001, Roche, Basel, Switzerland) of polyvinylidene difluoride (PVDF). Skim milk was used to cover the cell membranes before the membranes were exposed to primary antibodies (PCNA 13110, MMP2 40994, MMP9 13667, Actin 4970, Bax 5023, Bcl2 4223, Cleaved caspase3 9661, 1:1000, Cell Signaling Technology, Danvers, MA, USA; ki67 ab92742, FUT4 ab231561, 1:1000, Abcam, Cambridge, UK) for a whole night at 4°C. The following day, they were cleaned with Tris-buffered saline with Tween 20 (TBST) (T9039, Sigma-Aldrich, St. Louis, MO, USA). Next, they were incubated for 1 hour at room temperature with the corresponding secondary antibodies (Goat Anti-Rabbit IgG (H+L) Antibody 35401, 1:500, Cell Signaling Technology, Danvers, MA, USA). In addition, enhanced chemiluminescence (ECL) (32209, Thermo Fisher Scientific, Waltham, MA, USA) was adopted to explore immunoreactive bands, which were then photographed using image-processing software (NIH, Bethesda, MD, USA).

### 2.7 CCK-8 assay

First, 1×10^4^ cells/well of 96-well plates were injected with cells. Following cell adhesion, each well received 100 μL of media with 10 μL of CCK-8 (CK04, Dojindo Molecular Technology, Kumamoto, Japan). Make a recording of the optical density (OD) at 450 nm after 1 hour.

### 2.8 Colony formation assay

After 48 h after transfection, cells were collected and injected at the density of 200 cells/well in 6-well plates. Next, they were grown for two weeks in a complete medium. After one week, then every other week, switch the medium. Following the generation of the clone, the medium should be aspirated and washed twice with phosphate-buffered saline (PBS) (C0221A, Beyotime, Shanghai, China). After being fixed in methanol for 20 minutes, the cells were stained with 0.1% crystal violet (C0121, Beyotime, Shanghai, China) for 20 minutes. Then the cells were imaged to record colony development after three PBS washes.

### 2.9 EdU assay

Using the Cell-Light^TM^ EdU DNA Cell Proliferation Kit (C10313, RiboBio, Guangzhou, China), EdU assay was conducted to determine proliferation of cells. Transfected cells were exposed to 50 mM EdU and they were incubated for an additional 2 h after the incubation for 48 h at 37°C and 5% CO_2_. Next, the proliferating cells were stained with an Apollo dye solution after the cells had been fixed with 4% paraformaldehyde (P0099, Beyotime, Shanghai, China). All cells' nucleic acids were stained with DAPI (C1002, Beyotime, Shanghai, China). By the program ImageJ (Version 1.8.0; National Institutes of Health, Sacaton, AZ, USA), cell proliferation rates were estimated. By a fluorescent microscope, pictures were captured.

### 2.10 Wound-healing assay

Inoculated into 12-well plates were 2×10^5^ cells/well (three replicates for each group), which were then cultivated until fusion. To remove isolated cells, scrape the monolayer with the tip and then wash with a medium devoid of serum. Then, either with or without the addition of UCB-Exos (100 μg/well) as well as miR-21-3p inhibitor, cells were grown in a complete medium. HSF was captured on camera after 0, 12 and/or 24 h, whereas HMEC was captured on camera at 0, 6 and/or 12 h following an injury. The migration area (%) = (A0-An)/A0×100 was used to determine the wound closure area, where An is the residual wound area at the measurement point, and A0 is the original wound area.

### 2.11 Transwell assay

An 8 μm filter was used to inoculate 1×10^4^ cells/well (three replicates for each group) into the upper chamber of a 24-well transwell plate (3071834, Corning, NY, USA) that had a low-serum (5% FBS) medium. Subsequently, complete media (10% FBS) was used in the bottom chamber, either with or without the addition of UCB Exos (50 μg/well) and miR-21-3p inhibitor. 12 h later, the cells affixed to the filter membrane's upper side were washed. Morever, the migrating cells on the bottom surface were stained for a few of minutes with 0.5% crystal violet. Then under a light microscope (Leica DMI6000B, Germany), migration levels were seen.

### 2.12 TUNEL assay

Cells were raised twice in PBS before being fixed that kept for 15 minutes in 4% paraformaldehyde. Then they were permeabilized for 20 minutes in 0.25% Triton-X 100 (P0096, Beyotime, Shanghai, China). Carry out the TUNEL assay with reference to the manufacturer's guidances and recommendations (11767305001, Roche, Basel, Switzerland). In short, cells were treated with a click reaction mixture after being initially incubated at 37°C for 45 min in a reaction mixture which contained terminal deoxynucleotidyl transferase (TdT). Methyl green was used to stain the cell nuclei.

### 2.13 Statistical analysis

For all statistical studies, IBM's Statistical Products and Services Solutions (SPSS) 20.0 software (Armonk, NY, USA) was performed. All experiments involved in data presentation were repeated three times, and a mean ± standard deviation were employed to express experimental results. The t-test was conducted to make a comparison between two groups. Besides, one-way ANOVA or two-way ANOVA was conducted to make a comparison more than two groups. It was significant statistically at p < 0.05.

## 3. Results

### 3.1 Reduced expression of miR-371b-5p is observed in human osteosarcoma cells

**Figure 1:**
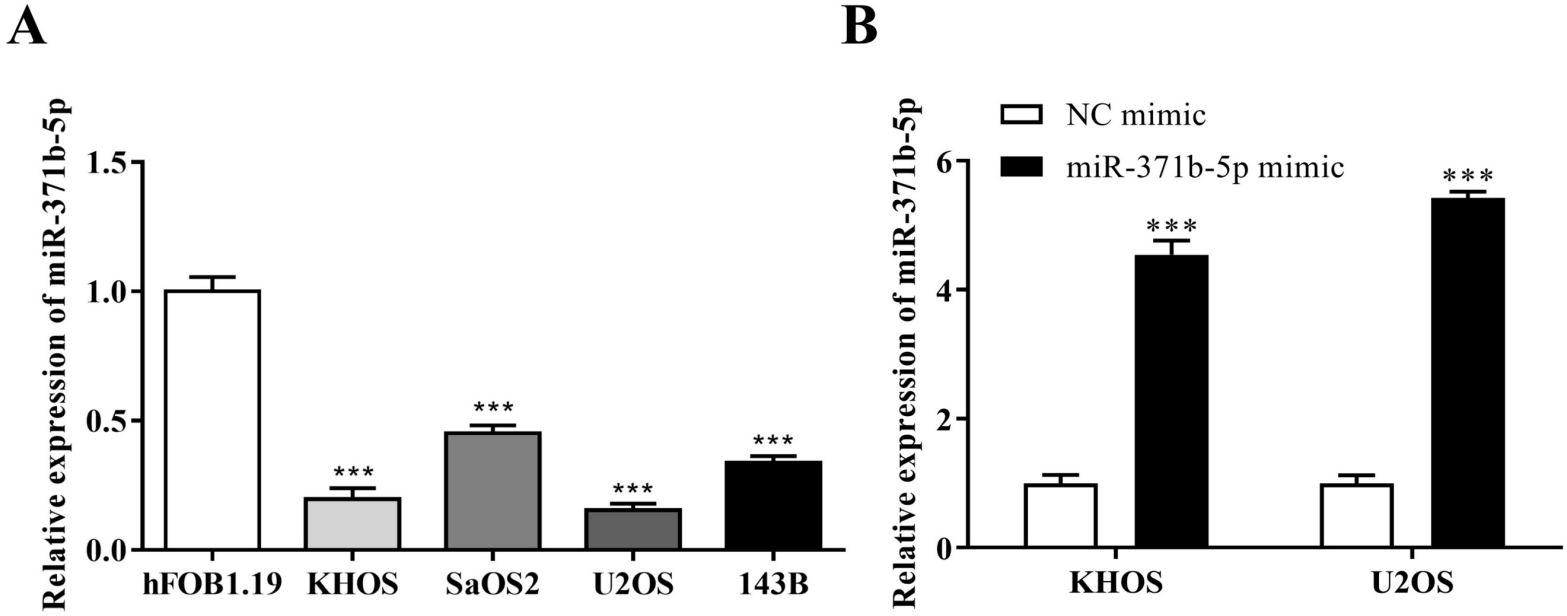
There was a downregulation of miR-371b-5p in human osteosarcoma cells. **A.** In comparision with the osteoblast cell line (hFOB1.19), according to the outcomes of qRT-PCR assay, the miR-371b-5p's expressions were considerably downregulated in human osteosarcoma cells KHOS, SaOS2, U2OS and 143B. **B.** MiR-371b-5p transfection replicated KHOS and U2OS miR-371b-5p levels in cells. ^***^p<0.001.

The expression of miR-371b-5p was reduced in human osteosarcoma cells KHOS, SaOS2, U2OS and 143B when compared with the osteoblast cell line (hFOB1.19) (*p* < 0.001, **Figure 1A**). KHOS and U2OS cell lines were then chosen for the further experiments for that miR-371b-5p was less expressed in KHOS and U2OS compared with SaOS2 and 143B. KHOS and U2OS cells were used to transfect the miR-371b-5p mimics. Besides, the transfection effectiveness was assessed by QRT-PCR assay. Then, after the transfection with miR-371b-5p mimics, the findings demonstrated that miR-371b-5p was greatly overexpressed in KHOS and U2OS cells (*p* < 0.001, **Figure 1B**).

### 3.2 miR-371b-5p prevents human osteosarcoma cells from proliferating, invading, and migrating

**Figure 2:**
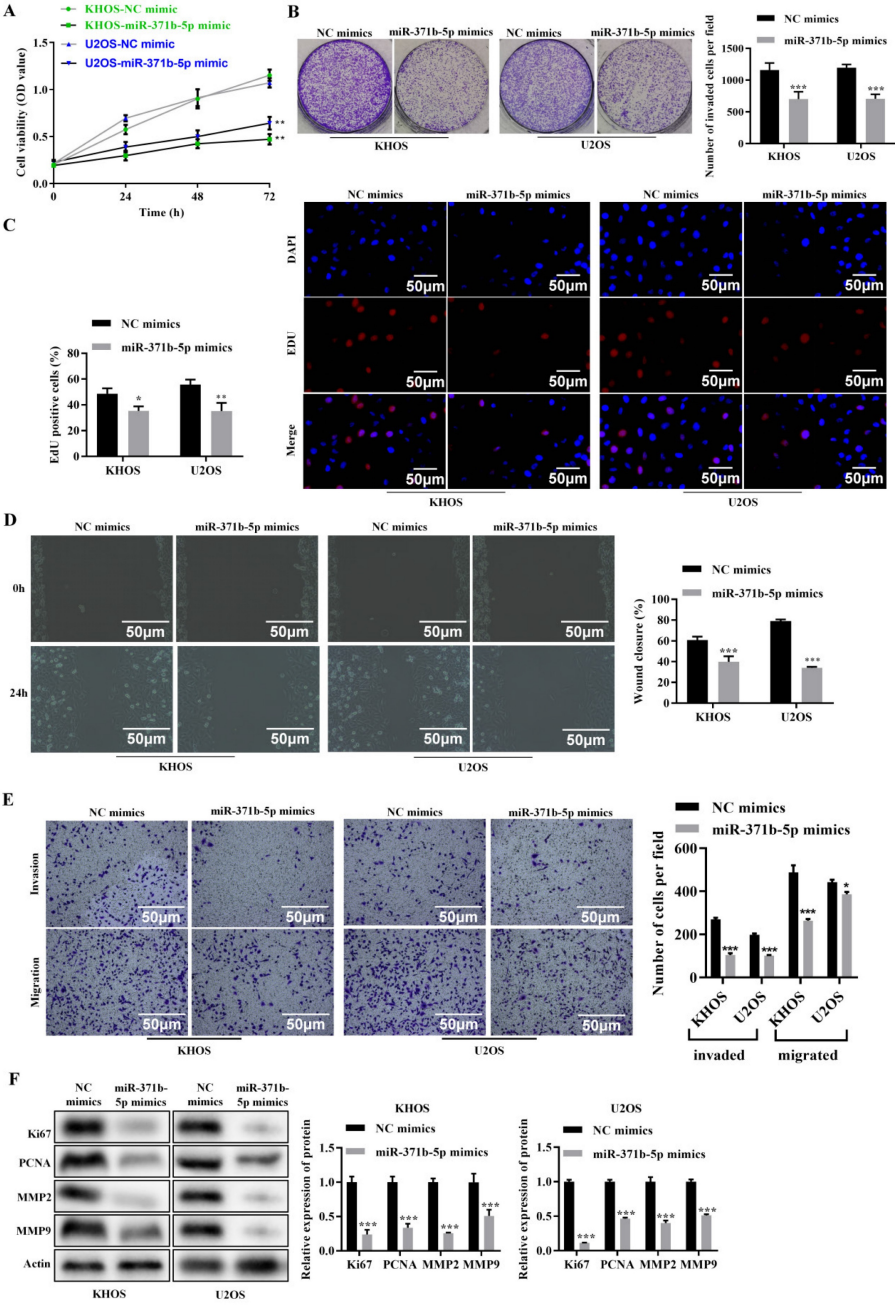
Human osteosarcoma cell's abilities to proliferate, invade and migrate are inhibited by miR-371b-5p. **A.** Overexpression of miR-371b-5p decreased the viability of cells in KHOS as well as U2OS cells. **B.** In KHOS and U2OS cells, miR-371b-5p mimics declined the quantity of colonies; example images are exhibited on the left, while the findings of the quantitative analysis are exhibited on the right. **C.** KHOS and U2OS cells were transfected with equivalent numbers of control mimics as well as miR-371b-5p mimics, and the EdU assay was carried out in these cells. Representative fluorescence images (200X) are exhibited on the left, while the outcomes of the quantitative analysis are shown on the right. **D.** The findings of the quantitative analysis are shown on the right, along with typical images from a wound-healing assay performed on KHOS as well as U2OS cells that were transfected with equal numbers of control mimics and miR-371b-5p mimics. The left shows typical photos (200X), and the right shows the outcomes of the quantitative analysis. **E.** KHOS and U2OS cell invasion and migration were markedly inhibited by miR-371b-5p. The left shows typical photos (200X), and the right shows the outcomes of the quantitative analysis. **F.** Reduced Ki67, PCNA, MMP2, and MMP9 expression is mimicked by miR-371b-5p. The control protein utilized was Actin. The findings of the relative quantitative analysis are shown on the right, while the representative photographs are exhibited on the left. ^*^p<0.05, ^**^p<0.01, ^***^p<0.001.

According to the CCK-8 assay, miR-371b-5p overexpression dramatically decreased the viability of human osteosarcoma cells (*p* < 0.01, **Figure 2A**). MiR-371b-5p mimics dramatically reduced the cell proliferation of human osteosarcoma cells, according to the colony formation assay (*p* < 0.001, **Figure 2B**). MiR-371b-5p mimic-transfected human osteosarcoma cells displayed a significant decrease in proliferation, according to the EdU assay (*p* < 0.05, **Figure 2C**). According to the wound-healing assay, miR-371b-5p overexpression severely reduced the migration of human osteosarcoma cells (*p* < 0.001, **Figure 2D**). Overexpression of miR-371b-5p substantially impeded the abilities to invade and migrate of human osteosarcoma cells, in terms of the findings of the Transwell assay (*p* < 0.05, **Figure 2E**). Ki67 and PCNA, the proliferation-associated proteins, as well as MMP2 and MMP9, the migration-associated proteins, were all markedly reduced by overexpression of miR-371b-5p (*p* < 0.001, **Figure 2F**).

### 3.3 miR-371b-5p promotes apoptosis in human osteosarcoma cells

**Figure 3:**
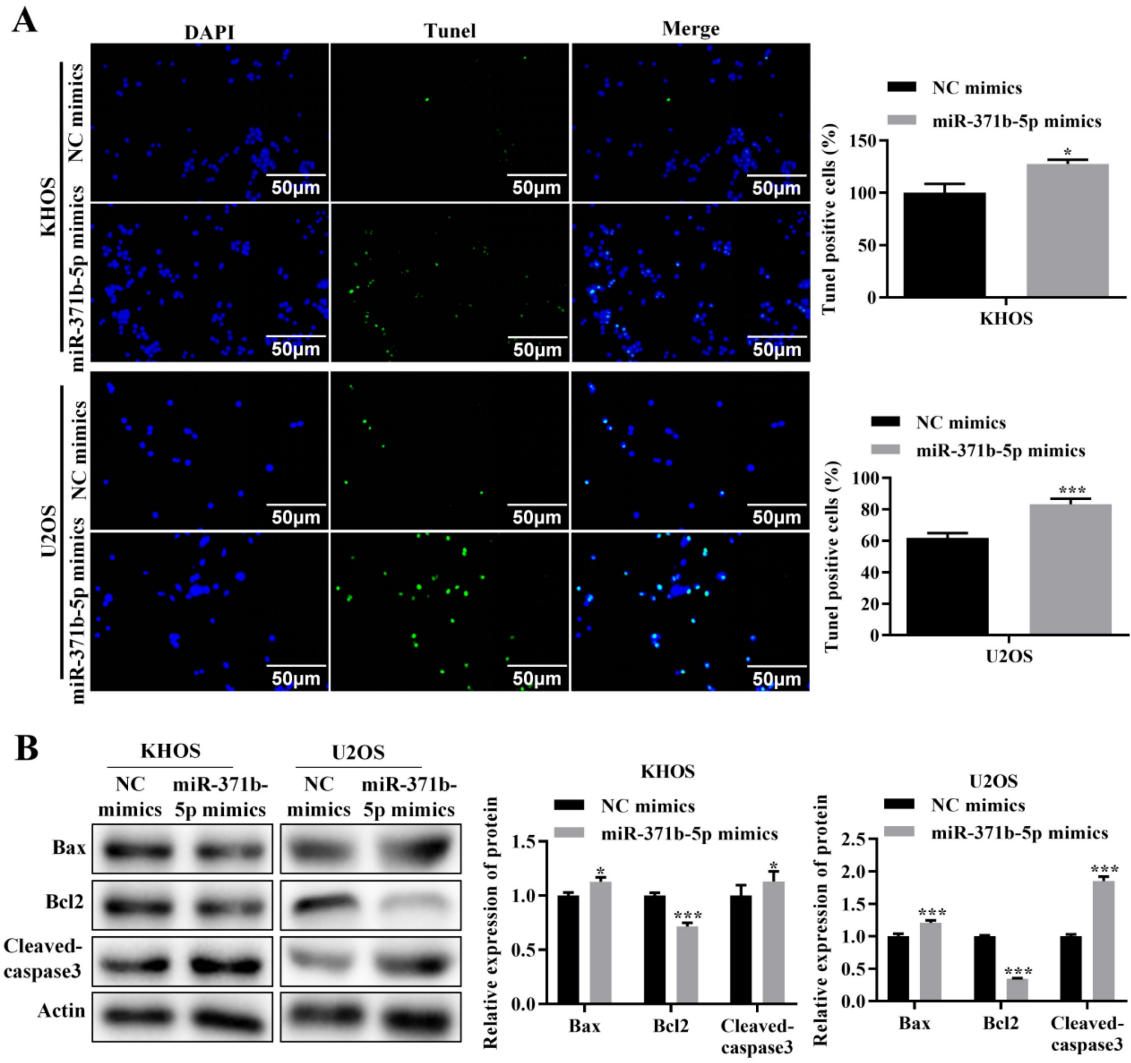
In human osteosarcoma cells, miR-371b-5p induced apoptosis. **A.** MiR-371b-5p overexpression increased apoptosis in KHOS as well as U2OS cells. The findings of the quantitative analysis are shown on the right, while typical fluorescence pictures are shown on the left (200X). **B.** Cleaved caspase3 and Bax expression were both elevated by miR-371b-5p mimics, whereas Bcl-2 expression was lowered. The protein utilized as a control was actin. On the left are representative photos, and on the right are the findings of a relative quantitative analysis. ^*^p<0.05, ^**^p<0.01, ^***^p<0.001.

TUNEL assay revealed that in human osteosarcoma cells, miR-371b-5p overexpression dramatically increased apoptosis (*p* < 0.05, **Figure 3A**). The pro-apoptotic proteins Bax and caspase3 were expressed considerably more when miR-371b-5p was overexpressed in U2OS cells, compared with the inhibitory apoptotic protein Bcl-2. Overexpression of miR-371b-5p in KHOS cells obviously declined the anti-apoptotic protein Bcl-2's expression, while they induced an increased trend in the expression of the apoptotic proteins Bax and caspase3 (*p* < 0.05, **Figure 3B**).

### 3.4 FUT4 is a target gene of miR-371b-5p

**Figure 4:**
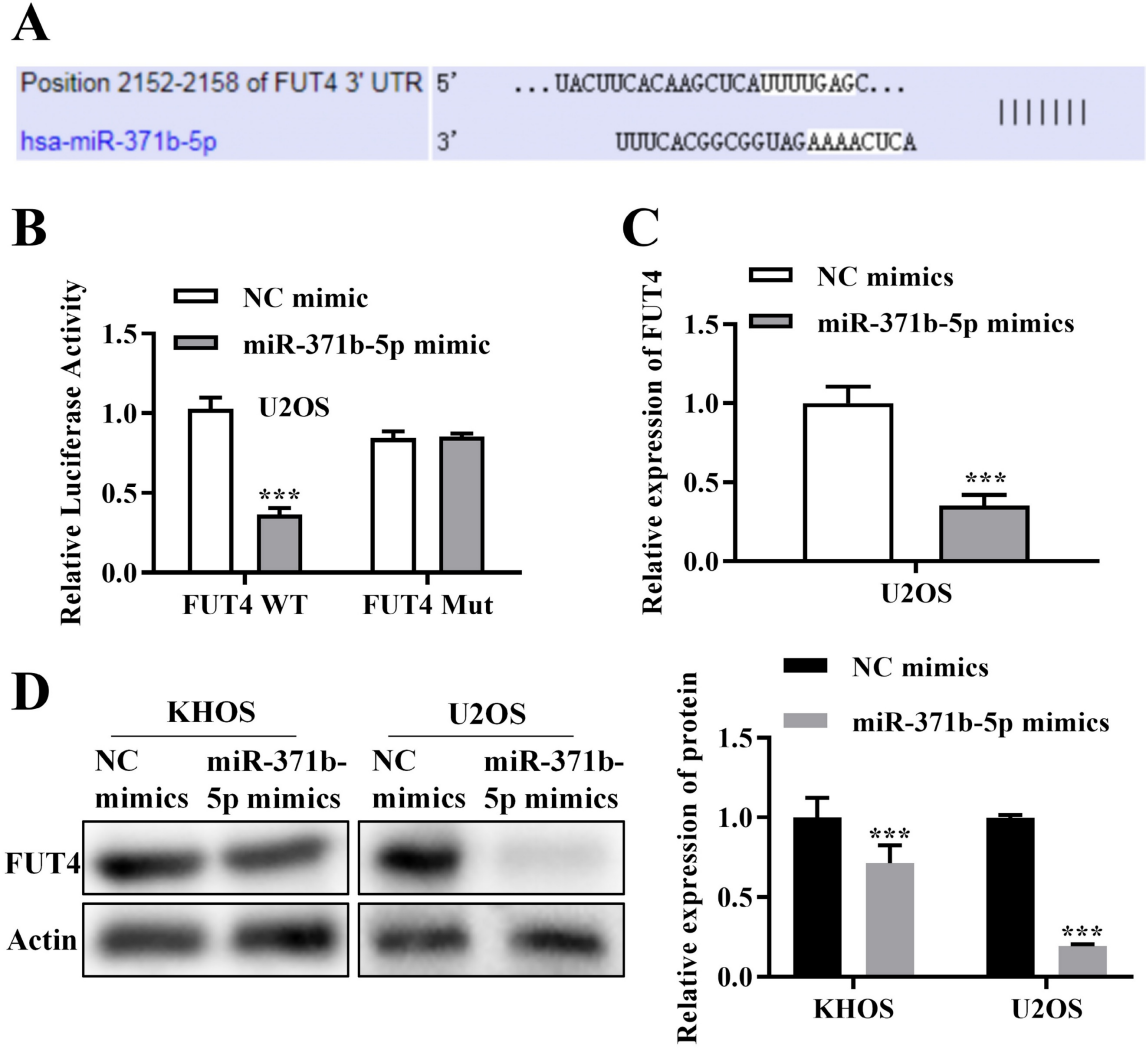
The miR-371b-5p target gene is FUT4. **A.** Possible sequence for merging miR-371b-5p with FUT4. **B.** When FUT4-WT and miR-371b-5p mimics were cotransfected into osteosarcoma cells, relative luciferase activity was markedly decreased. **C.** Transfection of miR-371b-5p mimicked down-regulation of FUT4 mRNA levels in U2OS cells. **D.** Transfection of miR-371b-5p mimicked down-regulation of FUT4 protein levels in KHOS and U2OS cells. Representative images are indicated on the left, while the findings of relative quantitative analysis are exhibited on the right. ^***^p<0.001.

Using bioinformatics analysis, it was hypothesized that FUT4 and miR-371b-5p would combine (**Figure 4A**). In U2OS cells, FUT4 WT and FUT4 MUT were built based on the combined sequences for dual-luciferase reporter analysis. The findings confirmed the relationship between FUT4 and miR-371b-5p by demonstrating that in U2OS cells cotransfected with FUT4-WT and miR-371b-5p mimics, the relative luciferase activity was drastically reduced (**Figure 4B**). The expression of FUT4 in U2OS cells was measured through QRT-PCR assay, and miR-371b-5p overexpressed U2OS cells showed obviously lower expressions of FUT4 than the control group (*p* < 0.001, **Figure 4C**). In KHOS and U2OS cells, FUT4 expression was assessed using a Western blot test, and it was clearly lower in the group that had miR-371b-5p overexpression (*p* < 0.001, **Figure 4D**).

### 3.5 miR-371b-5p mediates cell proliferation, invasion and migration of osteosarcoma through degradation of FUT4

**Figure 5:**
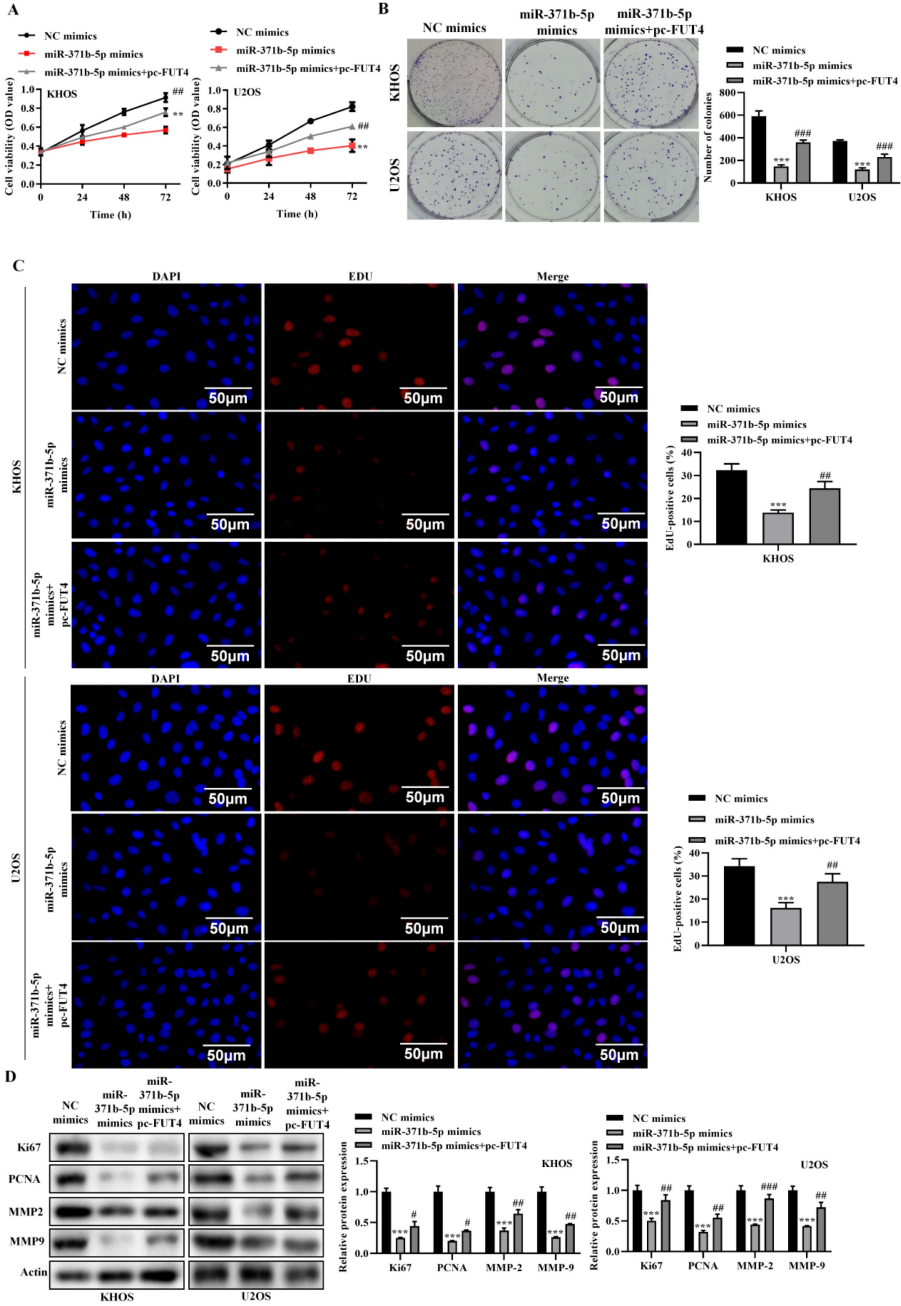
Overexpression of FUT4 partially reversal the inhibitory effects caused by miR-371b-5p on the proliferation, invasion and migration abilities of human osteosarcoma cells. **A.** CCK8 assay detected that pc-FUT4 could partially relieve the inhibitory effect of miR-371b-5p overexpression on cell viability. Pc-FUT4 was found to alleviate the effect of miR-371b-5p mimics on the proliferation ability of human osteosarcoma cells by **(B)** colony formation assay and **(C)** EdU assay (200X); the left indicates representative images, and the right indicates the results of quantitative analysis. The effects of transfection with miR-371b-5p mimics and pc-FUT4 on the migration and invasion capabilities of KHOS and U2OS cells were examined by **(D)** Overexpression of FUT4 partially reversed the miR-371b-5p-induced decrease in Ki67, PCNA, MMP2 and MMP9 expressions in KHOS and U2OS cells by western blot assay. The protein utilized as a control was Actin. On the left are representative photos, and on the right are the findings of the relative quantitative analysis. ^*^p<0.05, ^**^p<0.01, ^***^p<0.001 (miR-371b-5p mimics in comparison with NC mimics). ^#^p<0.05, ^##^p<0.01, ^###^p<0.001 (miR-371b-5p mimics compared with miR-371b-5p mimics+pc-FUT4).

**Figure 6:**
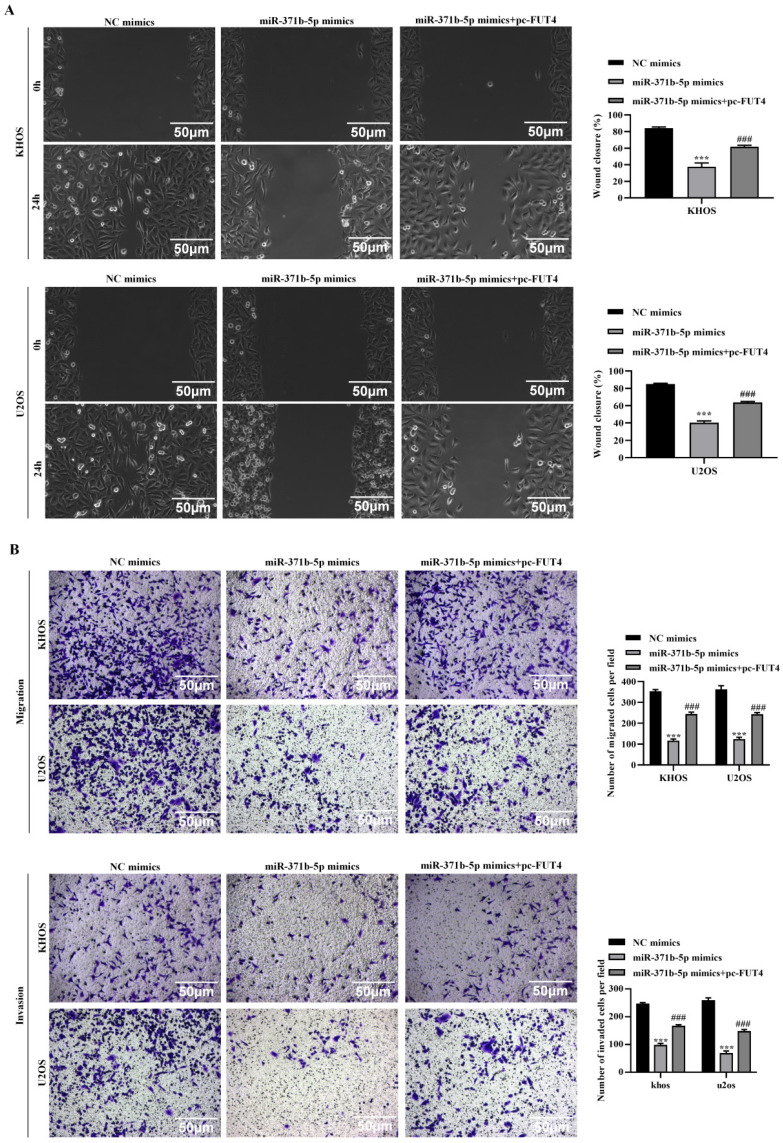
Overexpression of FUT4 partially reversal the inhibitory effects caused by miR-371b-5p on the proliferation, invasion and migration abilities of human osteosarcoma cells. The effects of transfection with miR-371b-5p mimics and pc-FUT4 on the migration and invasion capabilities of KHOS and U2OS cells were examined by **(A)** wound-healing assay and **(B)** transwell assay. Representative images are exhibited on the left (200X), while the findings of the quantitative analysis are exhibited on the right. ^*^p<0.05, ^**^p<0.01, ^***^p<0.001 (miR-371b-5p mimics in comparison with NC mimics). ^#^p<0.05, ^##^p<0.01, ^###^p<0.001 (miR-371b-5p mimics compared with miR-371b-5p mimics+pc-FUT4).

To further understand the molecular functions and consequences of miR-371b-5p/FUT4 on osteosarcoma, we conducted a variety of rescue experiments. In terms of the CCK-8 assay and colony formation assay, overexpression of FUT4 partially reversed the decline in cell viability which was induced by overexpression of miR-371b-5p in KHOS and U2OS cells (*p* < 0.01, **Figures 5A, 5B**). The EdU assay revealed that overexpressing FUT4 partly reversed the suppression of cell growth brought on by overexpressing miR-371b-5p (*p* < 0.01, **Figure 5C**). Inhibition of the production of the migration-associated and proliferation-associated proteins, MMP2 and MMP9, brought on by overexpression of miR-371b-5p was partially restored by overexpression of FUT4, according to the Western blot assay (*p* < 0.05, **Figure 5D**). The wound-healing assay revealed that overexpressing FUT4 partly corrected the inhibition of cell migration brought on by overexpression of miR-371b-5p (*p* < 0.001, **Figure 6A**). Transwell assat demonstrated that overexpressing FUT4 partly reversed the inhibiting ability of cell invasion and migration brought on by overexpressing miR-371b-5p (*p* < 0.001, **Figure 6B**).

### 3.6 miR-371b-5p mediates apoptosis in osteosarcoma through degradation of FUT4

**Figure 7:**
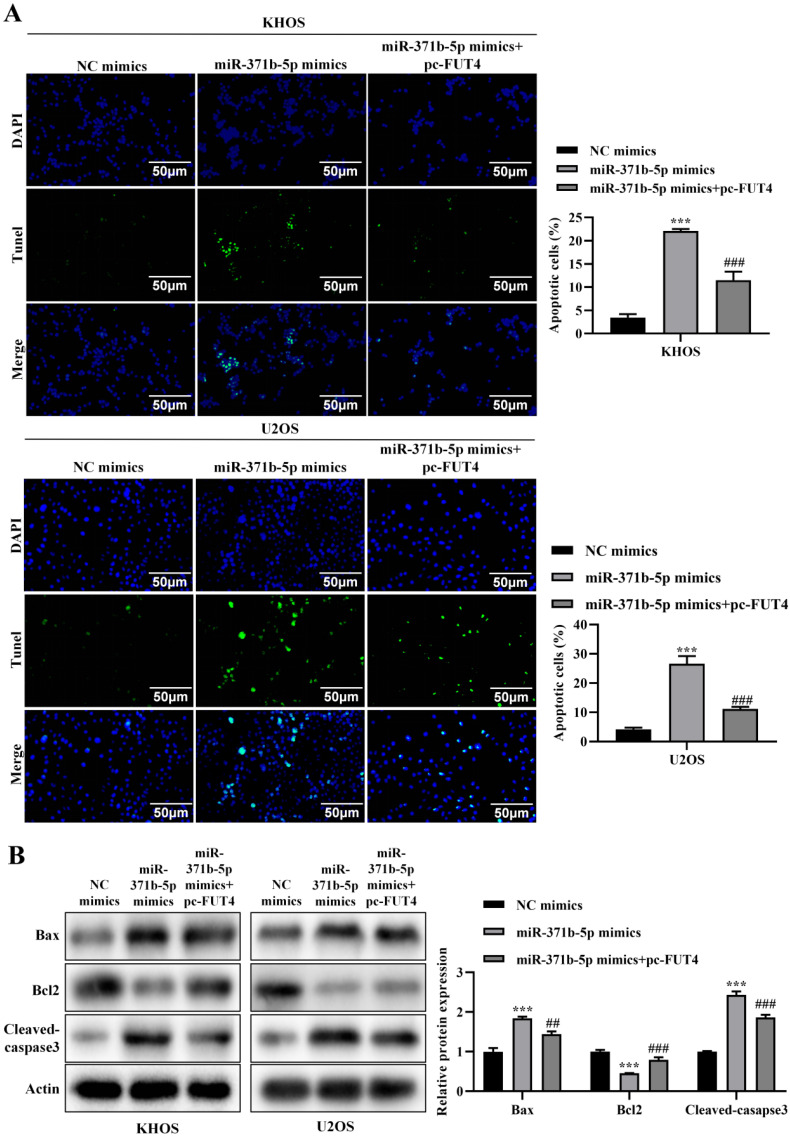
Overexpression of FUT4 could partially reverse the effect of miR-371b-5p on apoptosis of human osteosarcoma cells. **A.** TUNEL assay found that overexpressing FUT4 partially reversed the enhanced apoptosis caused by miR-371b-5p overexpression in KHOS and U2OS cells. Representative fluorescence pictures are shown on the left (200X), while the findings of quantitative analysis are shown on the right. **B.** Partial reversal of miR-371b-5p overexpression by FUT4 led to upregulation of Bax, Cleaved caspase3 expression as well as decreased Bcl-2 expression. Representative images are shown on the left, while findings of the relative quantitative analysis are shown on the right. ^*^p<0.05, ^**^p<0.01, ^***^p<0.001 (miR-371b-5p mimics were in comparison with NC mimics). ^#^p<0.05, ^##^p<0.01, ^###^p<0.001 (miR-371b-5p mimics were compared with miR-371b-5p mimics+pc-FUT4).

In KHOS and U2OS cells, overexpression of FUT4 partly corrected the increased apoptosis brought on by overexpression of miR-371b-5p, according to a TUNEL experiment (*p* < 0.001, **Figure 7A**). Western blot analysis revealed that overexpressing FUT4 partly reversed the rise in Bax and caspase3 expression and reduction in Bcl-2 expression brought on by overexpressing miR-371b-5p (*p* < 0.01, **Figure 7B**).

## 4. Discussion

A commonly involved bone tumor in children as well as teenagers is osteosarcoma 18. In recent years, there hasn't been much improvement in the prognosis of osteosarcoma. However, as genomics and miRNA research has advanced, the possible function of miRNA in carcinogenesis and progression has drawn increased attention, and the development of osteosarcoma has been a major source of concern 19. This study initially looked at the expressions of miR-371b-5p in osteoblast and osteosarcoma cells. By establishing a potential down-regulation of miR-371b-5p expression in osteosarcoma cells compared with osteoblasts, the findings revealed that miR-371b-5p have a possible impact on the formation of osteosarcoma. KHOS and U2OS cells were chosen as *in vitro* experimental models because miR-371b-5p expressions were considerably lower in these cells, further illuminating their biological activities. According to the findings, miR-371b-5p overexpression obviously reduced osteosarcoma cell's abilities to proliferate, invade and migrate while promoting osteosarcoma cell apoptosis. Previous mouse research proved the overexpression of miR-371b-5p in osteosarcoma tumor tissues when mice were infected with modified exosomes expressing the miR-371b-5p gene 10. Therefore, we proposed that miR-371b-5p controls cell's abilities to proliferate, invade, migrate and conduct apoptosis, which in turn drives osteosarcoma progression. Furthermore, recent studies have shown that the lncRNA/circRNA-miRNA-mRNA competitive endogenous RNA (ceRNA) network plays a crucial role in osteosarcoma drug resistance, although the underlying molecular mechanisms remain unclear 20, suggesting that future research can focus on the ceRNA involving miR-371b-5p and its role and mechanisms in osteosarcoma drug resistance.

The miRNA exerts its functions by degrading target genes 21. In this study, a bioinformatic approach was adopted to search for downstream targets of miR-371b-5p. FUT4 was ultimately screened. MiR-371b-5p and FUT4 were linked, and this was validated by a dual-luciferase reporter analysis. Previous researches have demonstrated that miR-125a-5p and lncRNA-MRUL interact to decrease the expression of FUT4 and hence increase osteosarcoma cell's ability to proliferate, migrate and invade 16. We thus put up the following hypothesis about FUT4's potential role in the growth and evolution of osteosarcoma. Additionally, in terms of QRT-PCR assay and Western blot assay, miR-371b-5p adversely influenced the relative level of FUT4 in osteosarcoma cells. Notably, overexpression of FUT4 largely overrode miR-371b-5p's reduction of osteosarcoma cell's ability to proliferate, migrate and invade as well as its augmentation of apoptosis. Thus, miR-371b-5p exerts a regulatory role in osteosarcoma through the downregulation of FUT4. Furthermore, studies have shown that low expression of miR-371b-5p in T-LBL upregulates the expression of Smad2/LEF1, subsequently increasing the expression of LINC00183, leading to progression and chemotherapy resistance 11. In triple-negative breast cancer, decreased expression of miR-371b-5p drives tumor progression through CSDE1/RAC1 regulation 12. Additionally, overexpression of miR-371b-5p in NSCLC promotes proliferation, migration, and invasion of non-small cell lung cancer cells via SCAI 13. Moreover, the miR-371b-5p/TFAP4 signaling pathway is involved in regulating senescence in colon cancer cells 22, while long non-coding RNA AC114812.8 promotes the progression of bladder cancer through the miR-371b-5p/FUT4 axis 17. These findings suggest that more downstream targets are potential areas for future research.

## Figures and Tables

**Table 1:** Primer sequences

Gene	Forward (5′-3′)	Reverse (5′-3′)
miR-371b-5p	ACTCAAAAGATGGCGGCACTTT	CTCTACAGCTATATTGCCAGCCAC
FUT4	AAGGTCCAGGCCCACTGAAG	CAGTTCAGGTGACAGAGGCTCA
GAPDH	ATGGGGAAGGTGAAGGTCG	GGGGTCATTGATGGCAACAATA
